# Parental positive regard and expressed emotion—prediction of developing attention deficit, oppositional and callous unemotional problems between preschool and school age

**DOI:** 10.1007/s00787-020-01625-1

**Published:** 2020-08-31

**Authors:** Ursula Pauli-Pott, Lotte Bauer, Katja Becker, Christopher Mann, Viola Müller, Susan Schloß

**Affiliations:** 1 Department of Child and Adolescent Psychiatry, Psychosomatics and Psychotherapy, Philipps-University of Marburg, Hans Sachs Str. 6, 35039 Marburg, Germany; 2 Center for Mind, Brain and Behavior (CMBB), University of Marburg and Justus Liebig University Giessen, Hans-Meerwein-Straße 6, 35032 Marburg, Germany

## Abstract

Parental expressed emotion and positive reinforcement are assumed to affect the development of oppositional and callous-unemotional behaviors in children at risk of attention deficit hyperactivity disorder (ADHD). As longitudinal research on this issue is scarce, we analyzed the respective links between preschool and school age. 138 five-year-old (*m* = 58.2, *s* = 6.2 months) children (59% boys) with elevated ADHD symptoms (according to screening) were assessed at the ages of five and eight years. At 5 years, maternal expressed emotion (using the Five Minute Speech Sample) and positive regard of child (using a standardized at-home observation procedure) were assessed. At 5 and 8 years, symptoms of ADHD, oppositional defiant disorder (ODD), and callous-unemotional (CU) behaviors were measured using a multi-informant approach. Multiple linear regression analyses revealed that positive regard specifically predicted a decrease in ODD symptoms between preschool and school age. The expression of high negative emotion specifically predicted an increase in CU behaviors. The development of ADHD symptoms was not predicted by parenting. Knowledge on these specific links can help to elaborate diagnostic and counseling processes in preschoolers with high ADHD symptoms. Underlying mechanisms and the role of neurocognitive deficits of the preschool child should be further analyzed.

## Introduction

There is broad evidence on an association between behavior problems in childhood and coercive, harsh parenting as well as low positive parental regard/reinforcement, and there is consensus on the notion that these parenting characteristics play an important role in the development of problem behaviors in childhood. In the present study, we focus on the developmental pathway from parenting to child behavior problems as one component of a more complex transactional process model in parent and child behavior development [1]. As outlined below, although many well-controlled studies have analyzed this pathway, it is not yet clear which psychopathological symptoms and components of problem behaviors might be affected by parenting characteristics.

In past decades, research focused on attention deficit hyperactivity disorder (ADHD), oppositional defiant disorder (ODD) and conduct disorder (CD), and the development of co-morbid ODD/CD in ADHD. More recently, so-called callous-unemotional (CU) traits have been distinguished within the ODD/CD spectrum, and regarded as a sub-dimension with specific etiology and developmental precursors. The three psychopathological dimensions, i.e. attention deficit/hyperactivity, oppositional, and CU symptoms are correlated and occur co-morbidly. In the course of development, however, ADHD symptoms often manifest first (mostly in preschool ages) and may be followed by the development of ODD/CD with or without CU behaviors [2–5]. The attention deficit problems might contribute to the development of the difficulties in emotion recognition and regulation in ODD/CD/CU [2, 6].

Regarding ADHD, several studies have investigated associations with parental “expressed emotion” (EE), i.e. parental criticism, warmth, and a positive relationship with the child, usually assessed by a short qualitative interview (the Five Minute Speech Sample, FMSS). It was assumed that the expression of high negative and low positive emotions towards the child indicate family stress, low parental support, low parental coping resources, and a negative parent–child relationship, which in turn affect the developmental course of the disorder including an increase in ADHD symptoms and the development of co-morbid ODD/CD [7–9]. Longitudinal research on these issues, however, is scare. Two longitudinal studies found associations between high maternal criticism and later ADHD. Peris and Baker [10] reported an association between criticism and ADHD at school age, while Musser et al., [7] reported that parental criticism was associated with continuously high ADHD symptoms across a two-year period in 7–11-year-olds. In both studies EE criticism comprised critical remarks and a negative description of the parent–child relationship. A third study, examining 5–18-year-olds diagnosed with ADHD, found no such associations across a six-year period [11]. To date, no studies have analyzed a potential prediction of change in ADHD symptoms by expressed emotion measures—e.g. the question of whether parental criticism and the description of a low positive relationship with the child predict change in ADHD symptoms over time.

Regarding ODD/CD and externalizing problems, associations with coercive/harsh and low positive parenting are well established. A recent meta-analysis found the strongest (medium-sized in clinical samples) cross-sectional associations between externalizing problems and “harsh control” including criticism and verbal violation/punishment [12]. In children with ADHD, Musser et al. [7] found high maternal criticism in a subgroup of children who showed increasing ODD symptoms. Similarly, in the study by Peris and Baker [10], maternal criticism was associated with later problem behaviors. Richards et al. [11], however, found no associations of maternal expressed emotions with later ODD/CD symptoms. In these studies, the prediction of change in oppositional symptoms was not analyzed. However, in the meta-analysis by Pinquart [12] small predictive effects of an increase in externalizing problems by parental harsh control and low positive regard were found in children from the general population.

Recent research has pointed to the relevance of the sub-dimension of CU traits with respect to the link between parenting and ODD/CD development. CU traits, i.e. lack of empathy, guilt or remorse, shallow or deficient affect, and limited prosocial emotions [13], may accompany ODD/CD in childhood. These characteristics are thought to show a comparatively high heritability, and to indicate an increased risk for the development of severe, chronic antisocial behavior. Therefore, CU traits have been assumed to be less susceptible to environmental conditions [14, 15]. However, estimates of heritability and shared environmental effects on CU traits seem to vary with age. In five-year-old twins, Tuvblad et al. [16] found medium-sized genetic and shared environmental effects.

Several longitudinal studies have analyzed the prediction of CU traits by parenting in high-risk aggressive as well as representative samples (see review by Waller et al. [17]). The studies converged in demonstrating longitudinal associations between negative/harsh parenting (reported by parent and/or child) and subsequently developing CU traits [17]. A poor parent–child relationship and harsh/negative parenting were found in children with early-onset chronic CU traits [18]. Two further longitudinal studies already analyzed parenting in infancy/early childhood in order to better capture the developmental origins of CU traits. Hyde et al. [19] followed more than 500 adopted children from 18 months onwards, assessing warmth and positive reinforcement from the adoptive mother through behavior observations at 18 months. The authors found that positive reinforcement predicted a decrease in CU traits until 27 months and, moreover, buffered the heritable risk due to antisocial behavior of the biological mother. Waller et al. [20] analyzed boys from low-income families. Low positive parenting (support, acceptance) at 24 months predicted CU behaviors at 42 months, which remained stable until the age of 10–12 years. In this study attention deficit and oppositional behaviors have been controlled. In a further longitudinal study, Wright et al. [21] found that parental low positive regard and sensitivity in infancy predicted CU behaviors at 2.5 and 5 years. Flom et al. [22] found contradictory results: In an investigation of CU behaviors and parenting in twins at two and three years of age, there was no prediction of change in CU behaviors by parenting, but significant inverse effects emerged, insofar as change in parenting was predicted by the CU behaviors of the child.

Taken together, the findings of many well-controlled longitudinal studies suggest that low positive regard/reinforcement and harsh parenting/criticism likely affect the development of psychopathological symptoms. However, several questions still remain open. As symptoms of ADHD and ODD often manifest for the first time in the preschool years and show relative instability until school age, they might be particularly malleable at this early stage, and parenting behaviors might play a significant role in this period. However, very few longitudinal studies have addressed this time period. Moreover, while ADHD symptoms often precede the development of ODD/CD/CU and may be involved in the psychopathological mechanisms of ODD/CD development, there is no longitudinal research on the predictive effect of these parenting characteristics in preschool children at risk of ADHD. Furthermore, it is possible that specific parenting components predict specific psychopathological processes, but only a small number of longitudinal studies have controlled for co-morbid symptoms. Therefore, it is not clear whether negative expressed emotion/criticism, low warmth and low positive regard/reinforcement precede the development of oppositional and CU behaviors in preschool children with elevated symptoms of ADHD. To the best of our knowledge, the present longitudinal study is the first to analyze these questions, i.e. the prediction of ADHD, ODD, and CU development by negative expressed emotion and low positive reinforcement in mother–child interaction in preschoolers with elevated ADHD symptoms.

We hypothesize that low positive regard/reinforcement and high criticism (i.e. critical comments and the description of a low positive relationship with the child) predict an increase in ODD and in CU symptoms between preschool and school age in preschool children with elevated ADHD symptoms. Moreover, we explore whether ADHD symptom development can be predicted by these parenting characteristics and whether specific parenting characteristics uniquely predict the development of a specific psychopathological dimension.

## Methods

### Participants

A sample of *n* = 138 five-year-old (*m* = 58.2, *s* = 6.2 months) children (*n* = 85, 59% boys) with elevated ADHD symptoms was recruited from child care centers. At recruitment, parents completed an ADHD screening questionnaire (FBB-ADHS-V *Döpfner, Görtz-Dorten, & Lehmkuhl* [23]*,*). Children who scored in the upper quartile (i.e. exceeded the lower bound of the 95% CI of the 75th percentile of the reference sample) were considered. Exclusion criteria from the study sample were IQ < 80, chronic diseases involving brain functions, any continuous pharmacological treatment, and insufficient German language skills of parent or child. Table 1 shows the descriptive data of the sample. 122 children took part in the 8-years assessment. The 16 (11.6%) children who dropped out of assessment did not differ from the remainder of the sample with respect to parent- and teacher-rated ADHD and ODD symptoms, age of child in months (*t*-scores between − 1.17 and 1.30), gender of child ($\chi$^2^ (1) = 0.29), and paternal education level ($\chi$^2^ (3) = 0.30). However, children who did not attend the 8-years assessment had mothers with a lower education level ($\chi$^2^ (3) = 8.48, *p* = 0.037). At 8 years, three children who had been diagnosed with ADHD in the interim were medicated with stimulants. In 41 cases, non-pharmacological interventions (e.g. parent counseling, psychotherapy, attention training) targeting the ADHD and/or externalizing symptoms of the child had been conducted. In the following analyses, we carefully controlled for the effects of these treatments (see below). Parents gave their written informed consent to participate in the study, and received an expense allowance of 50 Euros at the preschool assessment and 70 Euros at the school-age assessment. The study was approved by the Ethics Committee of the Medical Faculty, University of Marburg.

**Table 1:** Descriptive characteristics of the sample

Gender *n* (%)		
Male	85 (59.0)	
Female	63 (41.0)	
Age in months m(s)	58.2 (6.2)	
Education level	Of mother: *n* (%)	Of father: *n* (%)
Basic education	18 (13.0)	29 (21.0)
Work qualification	57 (41.3)	35 (25.4)
High school	21 (15.2)	29 (21.0)
College/university	42 (30.4)	41 (29.7)
(did not respond)		4 (2.9)
	5-years assessment	8-years assessment (*n* = 122)
ODD score *m* (s)	0.24 (1.06)	0.14 (0.96)
CU score *m* (s)	–	0.11 (0.94)
ADHD score *m* (s)	0.40 (0.89)	0.45 (1.88)
ADHD diagnosis *n* (%)		
Yes		29 (24)
No		93 (76)

*ADHD* attention deficit hyperactivity disorder, *ODD* oppositional defiant disorder, *CU* callous-unemotional

Sample: *n* = 138 children with elevated ADHD symptoms

### Procedure

At the 5-years assessment wave, data were collected within the scope of a home visit and a telephone interview with the mothers. During the home visit, mother–child interaction episodes were observed, the Five Minute Speech Sample (FMSS) was conducted with the mother, and the child underwent an intelligence test. At the 8-years assessment, a structured clinical interview was conducted with the mother at our lab while the child participated in neuropsychological tests. An investigator (psychologist) who was blind to all 5-years data of the child conducted the clinical interview with the mother. Parents and teachers completed questionnaires on ADHD symptoms, symptoms of ODD/CD, and CU traits.

### Variables

#### Assessments at the age of 5 years

*Parenting behaviors.* Positive reinforcement of the child by the mother was assessed by a standardized at-home observation procedure. Expression of negative emotion/criticism and perception of a positive relationship with the child were assessed by the respective subscales of the FMSS.

Maternal positive reinforcement was assessed in accordance with the procedure described by Hyde et al. [19]. Two standardized mother–child interaction episodes (working together on a puzzle, building a figure according to a template with a set of toy building blocks), each of 6 minutes duration, were observed. Positive reinforcement was coded during eight 30-s intervals (i.e. every 3rd interval) using the scale “praise” from the Family Interaction Observation System by Bertram et al. [24] (a German adaptation of the revised Family Observation Schedule (FOS-R-III) by Sanders et al. [25]. Praise is defined as the number of discernible positive, reinforcing utterances of the mother which are clearly directed to the child and clearly refer to a behavior or characteristic of the child. Inter-rater reliability was checked in 15% (*n* = 23) of cases (home visits conducted by two observers) and proved to be good (tau-b = 0.82). For further analyses, the scores of the 30-s intervals were summed up.

Maternal expressed emotion (EE) was assessed using the Five Minute Speech Sample (FMSS) by Magana et al. [26] in the modified version for preschool-aged children (PFMSS) by Daley et al. [27]. In the PFMSS, mothers are asked to talk about their thoughts and feelings towards their child for five minutes on an audiotape without any interruptions from the investigator. The audiotapes can be transcribed and coded regarding five components of expressed emotion, i.e. three global rating scales (initial statement, relationship, and warmth) and two frequency scales (number of positive comments, number of critical comments). The scales of the PFMSS proved to be valid and reliable [27, 28].

In the present study, we hypothesized that the expression of critical comments and the description of a low positive relationship by the parent predict the development of ODD and CU symptoms. As summarized in the introduction, previous research revealed significant concurrent and longitudinal associations specifically between these EE components and ADHD or externalizing symptoms. To test our hypotheses, we focus on the EE scales critical comments and relationship. For reasons of completeness, however, we also explored the concurrent and longitudinal associations of the other three EE scales (initial statement, warmth, and positive comments) with ADHD and externalizing symptoms.

In the present study, the tapes were transcribed and coded by a trained research assistant who was blind to the child’s ADHD and ODD symptom scores. Most mother–child relationships were coded as neutral (65%) or positive (33%) but seldom negative (2%). To avoid artifacts due to the skewed distribution, we created a dichotomous variable, i.e. distinguished between positive (scored with 1) and neutral/negative (scored with 0) relationships (EE-positive relationship). The frequency of critical comments (EE-critical comments) was used as a continuous variable. Inter-rater reliability was checked in 15% (*n* = 21) of cases and proved to be good (EE-positive relationship: Kappa = 0.85; EE-critical comments: ICC = 0.90). Regarding the initial statement, warmth and number of positive comments, inter-rater reliability was Kappa = 0.84, Kappa = 0.50, and ICC = 0.84, respectively.

*ADHD and ODD symptoms*. ADHD symptoms of the child were assessed by a structured clinical interview and by parent and teacher report. The ADHD scale of the Parental Account of Childhood Symptoms (PACS) interview [29] in the modified preschool version (Pre-PACS) [30] was conducted with the mother. To obtain the most precise descriptions of the child’s behavior, in this interview, parents are asked in a first step to report on defined behaviors of the child in specified situations in the last week. Based on this recall, in a second step, parents are asked to assess the intensity and frequency of the circumscribed symptoms [29] in the last three months. The preschool version of the PACS interview has demonstrated good psychometric properties, and proved suitable for ADHD diagnosis as well as the assessment of ADHD symptoms as a dimensional variable [31]. The ADHD scale of the interview shows good test–retest reliability (0.78, 15-week interval) and discriminates significantly between children with ADHD and healthy controls [31]. Parents and child-care teachers completed the ADHD rating scale (FBB-ADHS-V) of the “Diagnostic System for Psychiatric Disorders” (DISYPS-II) by Döpfner et al. [23]. The parent and teacher version have shown high internal consistency (Cronbach’s alpha: 0.94 and 0.93) and good validity (e.g. significant differentiation between children with and without an ADHD diagnosis [32]). In the present study, the three ADHD scores showed good concurrent and predictive validity: Correlation coefficients among the three ADHD scores were: *r* = 0.54, *p* < 0.001 (Pre-PACS and FBB-ADHS-V parent), *r* = 0.23, *p* < 0.05 (Pre-PACS and FBB-ADHS-V teacher), and *r* = 0.30, *p* < 0.01 (FBB-ADHS-V parent and teacher). The Pre-PACS ADHD score and the FBB-ADHS-V parent and teacher score were significantly associated with the 8-years ADHD diagnosis (*r* = 0.28, *p* < 0.01; *r* = 0.33, *p* < 0.001; *r* = 0.27, *p* < 0.01) and with the 8-years ADHD summary score (*r* = 0.32, *p* < 0.001; *r* = 0.46, *p* < 0.001; *r* = 0.31, *p* < 0.01). We created a summary score on the 5-years ADHD symptoms by adding up the z-transformed ADHD symptom scores (i.e. Pre-PACS ADHD score, parent and teacher questionnaire scores). Internal consistency was 0.54 (Cronbach’s Alpha). ODD symptoms were measured by the FBB-SSV questionnaire of the DISYPS-II diagnostic system. The questionnaire has shown good psychometric properties [32]. In the present study, the ODD symptom score (FBB-SSV) was significantly associated with the 8-years ODD and CU score (*r* = 0.30, *p* < 0.001; *r* = 0.34, *p* < 0.001).

#### Assessments at the age of 8 years

*ADHD symptoms and diagnoses*. At the age of 8 years, the ADHD diagnostic module of the Child and Adolescent Psychiatric Interview (CAPA, German version) by Angold et al. [33] was conducted with the mother. The CAPA is a well-validated, widely established clinical interview, which allows clinical diagnoses to be made according to the DSM-5. It has shown good correspondence with other established clinical interviews and questionnaires and discriminates well between clinical and non-clinical cases [34]. Of the 122 children, *n* = 29 (24%) received an ADHD research diagnosis. ADHD symptoms of the child were additionally assessed by parent and teacher questionnaires. Parents and school teachers completed the FBB-ADHS of the DISYPS-III [35]. The questionnaires capture ADHD symptoms according to ICD-10 and DSM-5 and have shown good psychometric properties, as reported above. Dimensional ADHD symptom scores were created by summing up the z-transformed scores of the parent and teacher ADHD scales (*r* = 0.70).

*ODD symptoms*. For the assessment of ODD symptoms, the conduct problems scale of the Strengths and Difficulties Questionnaire (SDQ) [36] was completed by the school teachers and the mothers. The SDQ is a widely used questionnaire with good psychometric properties [37, 38]. We created a dimensional ODD symptom score by summing up the z-transformed scores of the parent and teacher conduct problems scores (*r* = 0.43, *p* < 0.001).

*CU traits*. CU traits were assessed using the “prosocial behavior” scale of the SDQ and the “callous-unemotional” scale of the Antisocial Process Screening Device (APSD) [39]. Mothers and teachers completed the questionnaires. The items of the two scales have been proven to validly capture CU traits in 4–9-year-old children [40]. The CU scale of the APSD correlated significantly with ODD/CD symptoms and ADHD symptoms in 4–12-year-old children [40–42]. In the present study, the parent and teacher CU scores correlated significantly (*r* = 0.35, *p* < 0.001). We built a composite score by summing up the z-transformed scores.

*Treatment for ADHD and ODD/CU problems of child*. For purposes of control, mothers were asked whether the child, the parents or the family had received any non-pharmacological treatment (counseling, training, psychotherapy) targeting attention deficit or behavior problems of the child. If any treatment was reported, we recorded the number of sessions that had been received in the time period between the 5 and 8-years assessment.

### Data analysis

To facilitate the interpretation of the results of the multiple regression analyses, the bivariate correlations between the parenting measures and the psychopathological symptom scores at 5 and 8 years were calculated. These correlations and their significance levels have to be understood as descriptive statistics.

To test the first hypothesis, i.e. whether parenting characteristics predict an increase in ODD symptoms and CU behaviors between 5 and 8 years, hierarchical multiple regression analyses were conducted [43, 44], with the ODD and the CU score at 8 years as the criterion variables. In consecutive steps, control variables were introduced into the regression equations. To model “change in ODD symptoms between 5 and 8 years”, the 5-years ODD score was introduced into the regression analysis first, followed by the other control variables (i.e. gender of child and number of treatment sessions). The parenting scores were added in the final step. The change statistics reflect the predictive effect of the three parenting measures over and above the previously introduced control variables, i.e. the prediction of change in ODD symptoms between 5 and 8 years by the preschool parenting scores while controlling for gender and treatment. The β-coefficients of each parenting measure (in the final model) indicate the unique contribution to the prediction. In the case of a significant prediction by parenting, we explored (in a further regression model) whether the prediction by the parenting measures holds after additionally controlling for all ADHD and CU symptom scores.

An analogous procedure was used to test our hypothesis on CU behavior development. To explore the prediction of ADHD symptom development by the parenting characteristics, hierarchical multiple regression analysis was conducted. To explore whether the parenting scores predict the ADHD diagnosis, we conducted a logistic regression analysis. Hypotheses were tested with an alpha error of 5% (significance level of 0.05).

## Results

As shown in Table 2, the parenting measures correlated with concurrently (at 5 years) and subsequently (at 8 years) assessed ADHD, ODD, and CU symptoms. Specifically, low maternal positive regard in the mother–child interaction was significantly associated with concurrent ADHD symptoms and 8-years ODD symptoms (ODD: teacher and composite score). Maternal perception of a positive relationship (EE-positive relationship) with the child was negatively associated with 5- and 8-years ADHD, ODD, and CU symptoms (parent and composite scores). With the exception of ODD symptoms reported by the teacher at 8 years, maternal critical comments (EE-critical comments) were associated with ADHD, ODD, and CU symptom scores (CU: teacher, parent, and composite score).

**Table 2:** Correlations of 5-years parenting measures with 5- and 8-years ADHD-, ODD-, and CU scores

	5 y ADHD symptoms	5 y ODD symptoms	8 y ADHD diagnosis	8 y ADHD symptoms (p/t)	8 y ODD symptoms (p/t)	8 y CU symptoms (p/t)
Positive reinforcement	− .21*	− .01	− .16^+^	− .10 (− .13/− .06)	− .21* (− .14/− .28**)	− .04 (.00/− .09)
EE-positive relationship	− .27**	− .18*	− .20*	− .23* (− .26**/− .10)	− .22* (− .23*/− .10)	− .21* (− .28**/− .02)
EE-critical comments	.40***	.28***	.17^+^	.21* (.17^+^/.11)	.26** (.31***/.13)	.36*** (.33***/.22*)
Further PFMSS scales						
EE-initial statement	− .17^+^	− .15^+^	.08	− .06 (− .10/.08)	− .03 (− .12/.05)	− .12 (− .19*/− .02)
EE-positive comments	− .21*	− .15^+^	− .11	− .11 (− .16/.08)	− .15 (− .17^±^.03)	− .11 (− .12/-.06)
EE-warmth	.01	− .13	− .06	− .10 (− .06/-.09)	− .02 (− .08/.02)	− .09 (− .19^+^/.04)

*ADHD* attention deficit hyperactivity disorder, *ODD* oppositional defiant disorder, *CU* callous-unemotional, *EE* expressed emotion, *p* parent-report questionnaire, *t* teacher-report questionnaire

Significance: ^+^: *p* < .10, * *p* < .05, ** *p* < .01, *** *p* < .001

### Prediction of ODD and CU symptom development

The results of the hierarchical multiple regression analyses confirmed our hypotheses. ODD and CU symptom development was significantly predicted by parental low positive regard, EE-critical comments and EE-low-positive relationship (Table 3). Specifically, the three parenting measures together added significantly to the prediction of an increase in ODD symptoms between the age of 5 and 8 years while controlling for gender of child and treatment history (Table 3, model A1). This prediction remained significant after additionally adjusting for 5-years ADHD symptoms and 8-years ADHD and CU symptoms (Table 3, model A2). In both cases, low maternal positive regard/reinforcement significantly predicted increasing ODD symptoms over and above all other predictors. The three parenting measures together also predicted the development of CU symptoms while controlling for gender of child and treatment history (model B1, Table 3). This prediction remained significant after additionally controlling for the 5-years ADHD symptoms and the 8-years ADHD and ODD symptoms (model B2, Table 3). In both models (B1, B2), maternal high EE-critical comments predicted CU symptom development. In a next step, we controlled for pharmacological treatment of ADHD by excluding the three children who received medication. As shown in Table 3, the exclusion of these three children did not change any of the results.

**Table 3:** Prediction of ODD and CU symptoms by parenting measures

Multiple hierarchical linear regression analyses						
Model	Variables added	*R*_model_	*R*^2^_change_	*F*_change (df)_	*p*_change_	Model β-coefficients
A. Criterion: 8-years ODD symptoms						
A1	Positive reinforcement	.471** (.467**)^1^	.087 (.093)	3.671 (3.786)	.015 (.013)	− .21* (− .23*)
	EE-positive relationship					− .13 (− .13)
	EE-critical comments					.12 (.13)
A2	Positive reinforcement	.726*** (.724***)	.053 (.054)	3.520 (3.471)	.018 (.019)	− .23** (− .23**)
	EE-positive relationship					− .08 (− .09)
	EE-critical comments					.02 (.02)
B. Criterion: 8-years CU symptoms						
B1	Positive reinforcement	.503*** (.498***)	.072 (.074)	3.126 (3.097)	.029 (.030)	− .00 (− .01)
	EE-positive relationship					− .05 (− .04)
	EE-critical comments					.26** (.27**)
B2	Positive reinforcement	.724*** (.720***)	.048 (.049)	3.154 (3.056)	.028 (.032)	.11 (.11)
	EE-positive relationship					.03 (.03)
	EE-critical comments					.21* (.21*)

A1: controlling for: 5-years ODD symptoms, gender of child, treatment, A2: controlling for: 5-years ODD symptoms, 5-years and 8-years ADHD symptoms, 8-years CU traits, gender of child, treatment, B1: controlling for: 5-years ODD symptoms, gender of child, treatment, B2: controlling for: 5-years ODD symptoms, 5-years and 8-years ADHD symptoms, 8-years ODD symptoms, gender of child, treatment;

*ADHD* attention deficit hyperactivity disorder, *ODD* oppositional defiant disorder, *CU* callous-unemotional

Significance: (*): *p* < .10, * *p* < .05, ** *p* < .01, *** *p* < .001; ^1^in brackets: result after exclusion of the three medicated children

When exploring the prediction of ADHD symptom development by the parenting measures, we found that the parenting measures did not predict the development of ADHD symptoms between 5 and 8 years or an ADHD diagnosis at 8 years. Specifically, the complete prediction model of 8-years ADHD symptoms (including 5-years ADHD symptoms, gender of child, treatment history, and the three parenting scores) was significant at *R*_model_ = 0.610 (*p* < 0.001). The three parenting measures, however, did not contribute significantly to this prediction: *R*_change_ = 0.004, *F* = 0.223 (after exclusion of the medicated children: *R*_change_ = 0.006, *F* = 0.374). Similarly, the logistic regression analysis on the prediction of ADHD diagnosis at 8 years was statistically significant ($\chi$^2^ (6) = 26.4; *p* < 0.001; predictors: 5-years ADHD symptoms, gender of child, treatment history, and the three parenting scores) but the three parenting scores did not significantly contribute to this prediction ($\chi$^2^_change_ (3) = 3.19).

### Discussion

The present longitudinal study followed preschool children with elevated ADHD symptoms until school age, analyzing whether the development of ODD and CU symptoms can be predicted by parental positive regard/reinforcement and expressed emotion. Additionally, we explored the predictive effect of the parenting measures on ADHD symptom development. We found that an increase in ODD symptoms was significantly predicted by the parenting characteristics, and that maternal positive regard/reinforcement of the preschool child contributed significant unique variance to this prediction. The development of CU symptoms was also significantly predicted by the parenting characteristics, with the expression of negative emotion/criticism (EE-critical comments) making a unique and significant contribution to this prediction. In both cases, the findings did not change when adjusting for treatment history, gender of child and all co-morbid symptom dimensions. Thus, the respective parenting characteristics specifically predicted the ODD and CU dimension. Parenting did not predict any change in the ADHD symptoms of the child, and there was no significant prediction of the 8-years ADHD diagnosis by parenting when adjusting for the concurrent preschool ADHD symptoms.

To our knowledge, this is the first study to analyze the links between parenting and ODD as well as CU symptom development in preschool children with elevated ADHD symptoms. Our finding that ODD symptom development can be predicted by positive parental regard/reinforcement is in line with the results of Chronis et al. [45] and Hyde et al. [19]. In 4–7-year-old children with ADHD, Chronis et al. [45] found that positive parenting in highly demanding situations (but not low negative parenting or positive parenting in less demanding situations) predicted a decrease in oppositional symptoms. Moreover, Hyde et al. [19] found that positive reinforcement at 18 months predicted ODD symptoms in toddlers at 27 months. In children with ADHD symptoms, attention and neurocognitive deficits (e.g. low inhibitory control, high distractibility) may contribute to deficits in emotion recognition and regulation, which are thought to underlie ODD/CD development [46, 47]. Clearly discernable positive reinforcement directed to the preschool child might prevent this effect early in time by promoting social learning and self-regulation development [46, 48]. Positive parental regard, moreover, might strengthen a positive relationship with the parent and consequently the child’s compliance with parental demands [42].

While the study by Hyde et al. [19] also reported that positive reinforcement predicted CU symptom development, in the present study, we found no prediction of CU behaviors by observed maternal positive reinforcement. This discrepancy might lie in the differing ages of the children under study: CU behaviors might undergo critical development in toddlerhood and may be malleable to positive parenting primarily in this period [19]. Our finding that the development of CU behaviors was especially predicted by maternal expression of negative emotions/ high EE-critical comments is in line with other longitudinal studies in childhood [17]. In these studies, harsh, negative parenting predicted CU development in children from the general population and in children with elevated ODD/CD symptoms. Other studies analyzed the reverse pathway and found that CU traits of the child preceded negative parenting [22]. Transactional, cascading negative interaction processes may lead to increasing CU problems from the preschool years onwards [17]. Based on shared genetic predispositions (i.e. gene-environment correlation), the child’s behavior may elicit parental expression of high negative emotion, which in turn might contribute (via model and dysfunctional reinforcement learning) to the increasing development of low empathy and low prosocial emotions.

In the present longitudinal study, maternal negative expressed emotion (EE-critical comments) and description of a low positive relationship (EE-relationship) with the child did not predict an increase in ADHD symptoms or an ADHD diagnosis over and above concurrent symptoms of ADHD. To our knowledge, the prediction of change in ADHD symptoms by expressed emotion measures has not yet been analyzed. Nevertheless, our findings of concurrent and longitudinal associations of maternal critical remarks and description of a low positive mother–child relationship with the ADHD symptoms of the child correspond well to previous research, which reported that the expressed emotion measures were associated with concurrent, stabile ADHD symptoms [7, 10]. This might reflect primarily reverse associations, i.e. negative expressed emotion elicited by the child’s ADHD symptoms, a possibility which should be examined in future research.

Our study has several strengths, such as the longitudinal design, the use of a sample of preschool children with elevated ADHD symptoms, the use of a multi-informant approach to the measurement of the psychopathological symptoms of the child, the use of behavior observations in an at-home context for the assessment of positive reinforcement, the use of a validated version of the Five Minute Speech Sample for the assessment of expressed emotion, and the careful control for any previous and concurrent pharmacological and non-pharmacological treatment. However, some limitations of the study should also be mentioned. First, we cannot rule out the possibility that gene-environment correlation effects underlie the results, i.e. shared genes might be responsible for the mother’s and the child’s behaviors. However, the longitudinal design of the study does provide insights into the developmental succession. The predictive effects indicate possible environmental effects, which need to be confirmed in future research, e.g. by adoption studies. Second, as recommended [17], CU behaviors were measured at 8 years using a multi-informant approach, but were not assessed at the first assessment wave. At 5 years, we only assessed ODD and ADHD symptoms, and therefore failed to capture unique CU symptom variance (i.e. variance not shared with ODD or ADHD at 5 years). However, ODD and CU behaviors are closely associated in preschool-age [49]. Moreover, in the present study, 5-years ODD and ADHD scores significantly correlated with 8-years CU symptoms (0.34, *p* < 0.001, and 0.23, *p* = 0.013 respectively). Therefore, we can assume that by adjusting for the ODD and ADHD symptoms at 5 years, considerable and meaningful 5-year CU variance was controlled for. Third, we have not yet assessed pathways from 5-years symptom dimensions to 8-years parenting. Although the additional analysis of these pathways would not alter the present results, such analyses would provide more complete information. In the future, analyses of the complete cross-lagged processes should be conducted. Fourth, we did not calculate the inter-rater reliability for the ADHD scores of the clinical interviews. However, all interviewers underwent careful training and supervision. The significant concurrent and longitudinal associations between the ADHD interview and questionnaire scores indicate good validity of the measurement of ADHD in the present study.

To conclude, our longitudinal study revealed specific associations between parenting and behavior problem development in preschool children with elevated ADHD symptoms until school age. Maternal positive reinforcement likely buffered against and led to decreasing ODD symptom development. The development of CU behavior was preceded by the mother’s high negative expressed emotion. These links were specific for the symptom dimensions. Underlying mechanisms and the contribution of neurocognitive deficits should be further analyzed. The results can help to elaborate diagnostic and counseling processes in preschoolers with high ADHD symptoms.
